# Effect of propofol on oxidative stress status in erythrocytes from dogs under general anaesthesia

**DOI:** 10.1186/1751-0147-54-76

**Published:** 2012-12-26

**Authors:** Jae Yeon Lee, Myung Cheol Kim

**Affiliations:** 1 Department of Veterinary Surgery, College of Veterinary Medicine, Research Institute of Veterinary Medicine, Chungnam National University, Daejeon, 305-764, Korea

**Keywords:** Anaesthesia, Antioxidant effects, Dogs, Oxidative stress, Propofol

## Abstract

**Background:**

Alterations of the normal redox balance might be attributed to increase of plasma free-radical concentration and a disruption of the antioxidant defense system. One of the adverse effects of general anaesthetics is the exogen sources of reactive oxygen radicals that are responsible for several diseases. The purposes of the current study were to evaluate the effect of propofol on oxidative stress and to compare the differences between propofol induction only and induction plus continuous infusion on antioxidant status in dogs.

**Findings:**

Beagle dogs were evaluated in the present study. The dogs were assigned randomly to receive three treatments in a crossover model. The three treatments were: group 1 (n = 9), 2% isoflurane; group 2 (n = 9), anaesthesia induced with an intravenous (IV) bolus dose of 6 mg/kg propofol and maintained with 1.5–2% isoflurane; group 3 (n = 9), total IV anaesthesia (induction with 6 mg/kg propofol, infusion with 0.6 mg/kg/min propofol). The results of this study show that dogs exposed to isoflurane had decreased antioxidant enzymes activities, whereas dogs injected with propofol had increased antioxidant enzymes activities.

**Conclusions:**

The results of this study showed that an infusion dose of propofol has antioxidant effects in dogs. These effects may be beneficial to patients in whom free radicals play a role in oxidative stress, such as those with ischemia. Further studies are needed to evaluate whether these antioxidant effects of the anaesthetic are of clinical value.

## Findings

Oxidative stress is as an imbalance between the production of free radicals or reactive oxygen species (ROS) and antioxidant defense and is a major factor for postoperative morbidity and mortality [1]. Although various factors are implicated, the type and the duration of anaesthesia can alter the antioxidant defense system that influences oxidative stress. Certain anaesthetics have been attracting attention as a way to protect against the pathological states associated with oxidative stress [2]. Propofol (2, 6-diisopropylphenol) is an anaesthetic drug that has been widely-used intravenously in veterinary clinics. Propofol is chemically similar to the endogenous antioxidant α-tocopherol (vitamin E) and, theoretically, should have similar properties [3]. However, the oxidant and antioxidant status of propofol in dogs has not been fully evaluated. The purposes of the current study were to evaluate the effect of propofol on oxidative stress and to evaluate the dose response of propofol on antioxidant status in dogs.

Nine beagle dogs (age, 2–3 years; weight, 9–12 kg; four males and five females) were used. The experimental and housing protocols were approved by the CNU Animal Care and Use Committee (approval no. 2010-3-15). The dogs were assigned randomly to receive three treatments in a cross-over model. A wash-out period of at least 1 month was allowed between treatments. The three treatments were: group 1 (n = 9), 2% isoflurane; group 2 (n = 9), anaesthesia induced with an intravenous (IV) bolus dose of 6 mg/kg propofol, maintained with 1.5–2% isoflurane; group 3 (n = 9), total IV anaesthesia (induction with 6 mg/kg propofol and infusion with 0.6 mg/kg/min propofol). Heart rate, respiratory rate, rectal temperature, and saturation of peripheral oxygen were monitored continuously using a Pulscan-Component physiological monitor (Scionic, Seoul, Korea). After 60 min, the anaesthetics were discontinued and animals were allowed to recover.

Blood samples were collected via venipuncture from the jugular vein at each designated time (T1, before anaesthesia; T2, end of anaesthesia; T3, 24 h after anaesthesia) to assess oxidative stress status. Heparinized blood was obtained on the day of the experiment. After separating the erythrocytes from the plasma and the buffy coat by centrifugation for 5 min at 4000 rpm, the erythrocytes were lysed in four times their volume in ice-cold HPLC-grade water. This sample was centrifuged at 10,000 × g for 15 min at 4°C and the supernatant was immediately collected and stored at -80°C until measurement. Superoxide dismutase (SOD), catalase (CAT), and glutathione peroxidase (GPx) concentrations were measured with a commercial kit (Cayman Chemical Company, Ann Arbor, MI, USA) using a spectrophotometer (Bio-Tek Instruments, Winooski, VT, USA). All statistical analyses were performed using SPSS version 18.0 (Chicago, IL, USA). Results are expressed as mean ± SD. A Mann–Whitney *U*-test was applied with a p-value < 0.05 being considered significant.

**Figure 1:**
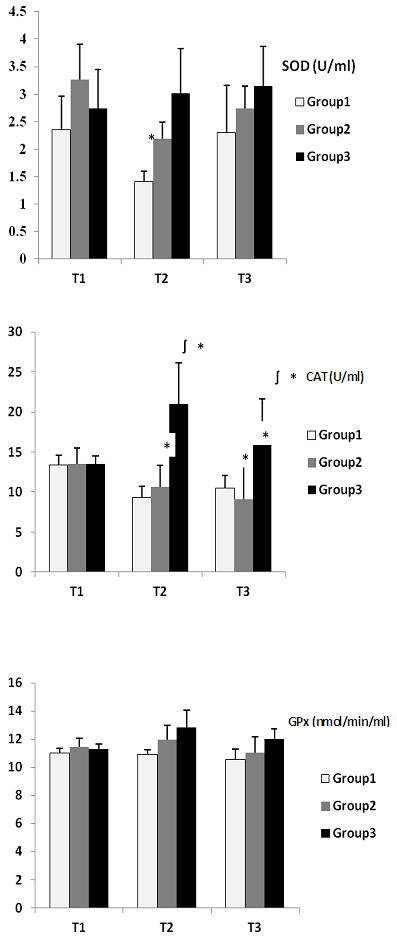
**Superoxide dismutase (SOD), catalase (CAT) and glutathione peroxidase (GPx) data in erythrocytes from dogs under anaesthesia.** Data are expressed as the mean ± SD (n = 9). Group 1, 2% isoflurane; group 2, anaesthesia induced with an intravenous (IV) bolus dose of 6 mg/kg propofol, maintained with 1.5–2% isoflurane; group 3, total IV anaesthesia (induction with 6 mg/kg propofol and infusion with 0.6 mg/kg/min propofol). * Significantly different (P < 0.05) from T1. ^∫^ Significantly different (p < 0.05) from Group 1. T1; before anaesthesia, T2; end of anaesthesia, T3; 24 h after anaesthesia.

All dogs were hemodynamically stable throughout the experiment. There was not any complication or technical problem related to the anaesthesia. In addition, all dogs were similar pattern in depth of anaesthesia and there were no significant worsening in heart rate, mean arterial pressure, rectal temperature, end-tidal carbon dioxide, peripheral oxygen saturation, and respiratory rates (Table 1). The SOD, CAT, and GPx data are summarized in Figure 1. SOD activity decreased significantly from baseline to T2 in groups 1 (p = 0.015) and 2 (p = 0.018). CAT activity decreased significantly from baseline to T2 (p = 0.028) and T3 (0.023) in group 1 and T2 (p = 0.026) and T3 (0.02) in group 2, whereas that of group 3 increased significantly from baseline to T2 (p = 0.018) and T3 (0.015). Significant differences were observed in CAT activities between groups 1 and 3 at T2 (p = 0.005) and T3 (0.008). Although no significant changes in GPx activities were observed, GPx activity tended to increase with time in group 3, whereas it tended to decrease in groups 1 and 2.

**Table 1:** **Heart rate (HR), mean arterial pressure (MAP), rectal temperature (RT), respiratory rate (RR), saturation of peripheral oxygen (SpO**_**2**_**) and end-tidal CO**_**2 **_**(ETCO**_**2**_**) in dogs**

**Parameter**	**Group**	**Pre**	**15 min**	**30 min**	**45 min**	**60 min**
HR (breath/min)	Group 1	109 ± 14	99 ± 16	93 ± 15	96 ± 15	91 ± 16
	Group 2	119 ± 21	116 ± 20	109 ± 11	110 ± 18	101 ± 12
	Group 3	99 ± 11	120 ± 18	108 ± 16	91 ± 18	96 ± 15
MAP (mmHg)	Group 1	85 ± 9	84 ± 5	85 ± 10	74 ± 12	81 ± 8
	Group 2	83 ± 11	86 ± 6	87 ± 12	81 ± 11	82 ± 9
	Group 3	78 ± 16	83 ± 7	83 ± 14	80 ± 16	80 ± 15
RT (°C)	Group 1	38.5 ± 0.4	38.3 ± 0.2	38.0 ± 0.4	37.5 ± 0.2	37.2 ± 0.2
	Group 2	38.8 ± 0.6	38.2 ± 0.4	38.0 ± 0.4	37.6 ± 0.2	37.2 ± 0.2
	Group 3	39.2 ± 1.1	39.0 ± 1.0	38.7 ± 0.8	38.2 ± 0.8	37.9 ± 1.0
RR (breath/min)	Group 1	27 ± 14	15 ± 11	11 ± 9	15 ± 7	13 ± 6
	Group 2	28 ± 11	25 ± 13	14 ± 8	15 ± 5	12 ± 10
	Group 3	30 ± 6	25 ± 10	11 ± 7	10 ± 6	11 ± 4
ETCO_2_ (mmHg)	Group 1	44.3 ± 10.14	47.3 ± 9.11	48.5 ± 11.17	55.7 ± 11.86	51.7 ± 13.68
	Group 2	48.5 ± 15.36	54.2 ± 11.23	51.6 ± 11.03	49.9 ± 9.01	48.6 ± 8.74
	Group 3	51.1 ± 11.24	51.3 ± 9.81	50.5 ± 9.16	54.7 ± 10.96	51.1 ± 14.18
SpO_2_ (%)	Group 1	99 ± 1	100 ± 0	100 ± 0	99 ± 1	100 ± 0
	Group 2	99 ± 1	100 ± 0	99 ± 1	100 ± 0	100 ± 0
	Group 3	99 ± 1	100 ± 0	100 ± 0	100 ± 0	99 ± 1

Data are expressed as the mean ± SD (n = 9).

Variables were measured before induction of anaesthesia (pre) and at 15, 30, 45, and 60 minutes after induction of anaesthesia.

The main enzymes that control the biological effects of ROS are SOD, CAT and GPx. SOD catalyses dismutation of the superoxide anion into H_2_O_2_; CAT detoxifies H_2_O_2_; GPx oxidizes reduced glutathione, inactivates H_2_O_2_, and reduces organic peroxides to their alcohols [4]. These antioxidant enzymes respond to increased oxidative stress and act as ROS scavengers. Therefore, measuring antioxidant enzymes activities reflects the antioxidative status of the antioxidant defense system.

In this study, the results show that dogs exposed to isoflurane had decreased SOD, CAT and GPx activities in erythrocytes. Conversely, animals injected with propofol had increased SOD, CAT and GPx activities. These results indicate that propofol is capable of increasing the antioxidant capacity in dogs. The previous our study showed that propofol has some benefit on overall oxidative stress status in dogs undergoing surgery [5].

The oxidative stress was multifactorial in origin; the main impacts were from the effects of the anaesthetic and from hemodynamic changes due to general anaesthesia. One of the factors that might be responsible for oxidative stress associated with isoflurane anaesthesia is increased proinflammatory cytokine expression in alveolar macrophages, which occurs after inhalation of volatile anaesthetics [6].

De La Cruz et al. (1999) found a similar antioxidant effect of propofol between 1-hour infusion and an initial bolus dose in humans [7]. However, in our study, the ability of propofol to reduce oxidative stress was documented, particularly in the propofol total IV group. Anaesthesia induced with an IV bolus dose of 6 mg/kg propofol showed no antioxidant ability, similar to the isoflurane group. In comparison the maximum increases rate in SOD, catalase and GPx activities, were 40%, 55.4%, and 14.8% respectively, between the isoflurane and propofol total IV groups. Poor recovery from anaesthesia is associated with increased oxidative stress. Moreover, it could be a main factor in postoperative mortality. Therefore, using drugs that have antioxidant or antiinflammatory properties might be useful to reduce oxidative injury.

In conclusion, the result showed that an infusion dose of propofol has antioxidant effects in dogs. These effects may be beneficial to patients in whom free radicals play a role in oxidative stress, such as those with ischemia. Further studies are needed to evaluate whether these antioxidant effects of the anaesthetic are of clinical value.

## Competing interests

The author does not have a financial or personal relationship with other people or organizations that could inappropriately influence or bias the content of the paper.

## Authors’ contributions

JYL designed and coordinated the study. She performed measurements of physiological parameters. MCK had the major responsibility for writing and finalising the manuscript. He performed statistical analysis. All authors participated actively in the writing of the manuscript, and all read and approved the final manuscript.

## References

[B1] Sies H Oxidative stress: oxidants and antioxidants Exp Physiol 1997 82 291 295 9129943 10.1113/expphysiol.1997.sp004024

[B2] Kato R Foëx P Myocardial protection by anesthetic agents against ischemia-reperfusion injury: an update for anesthesiologists Can J Anaesth 2002 49 777 791 10.1007/BF03017409 12374705

[B3] Aarts L van der Hee R Dekker I de Jong J Langemeijer H Bast A The widely used anesthetic agent propofol can replace alpha-tocopherol as an antioxidant FEBS Lett 1995 2 83 85 8001686 10.1016/0014-5793(94)01337-z

[B4] Michels C Raes M Toussaint O Remacle J Importance of Se-glutathione peroxidase, catalase, and Cu/Zn SOD for cell survival against oxidative stress Free Radic Biol Med 1994 17 235 248 10.1016/0891-5849(94)90079-5 7982629

[B5] Lee JY Oxidative stress due to anesthesia and surgical trauma and comparison of the effects of propofol and thiopental in dogs J Vet Med Sci 2012 74 663 665 10.1292/jvms.11-0221 22198056

[B6] Kotani N Hashimoto H Sessler DI Yasuda T Ebina T Muraoka M Matsuki A Expression of genes for proinflammatory cytokines in alveolar macrophages during propofol and isoflurane anesthesia Anesth Analg 1999 89 1250 1256 10.1213/00000539-199911000-00032 10553845

[B7] De La Cruz JP Zanca A Carmona JA de la Cuesta FS The effect of propofol on oxidative stress in platelets from surgical patients Anesth Analg 1999 89 1050 1055 10512289 10.1097/00000539-199910000-00043

